# BMSC-Derived Exosomes Alleviate Intervertebral Disc Degeneration by Modulating AKT/mTOR-Mediated Autophagy of Nucleus Pulposus Cells

**DOI:** 10.1155/2022/9896444

**Published:** 2022-07-09

**Authors:** Quan Xiao, Zhe Zhao, Yun Teng, Lungang Wu, Jinlong Wang, Hongjun Xu, Sumei Chen, Quan Zhou

**Affiliations:** ^1^Trauma Center, The Affiliated Lianshui County People's Hospital of Kangda College of Nanjing Medical University, Huai'an, Jiangsu Province 223400, China; ^2^Department of Orthopaedics, The Affiliated Huai'an Hospital of Xuzhou Medical University, Huai'an, Jiangsu Province 223002, China; ^3^Department of Orthopaedics, Xuyi People's Hospital, Kangda College of Nanjing Medical University, Huai'an, Jiangsu Province 211700, China; ^4^Department of Orthopaedic Surgery, The First Affiliated Hospital of Soochow University, Suzhou, Jiangsu, China; ^5^Department of Orthopaedics, The Affiliated Lianshui County People's Hospital of Kangda College of Nanjing Medical University, Huai'an, Jiangsu Province 223400, China

## Abstract

The pathogenesis of intervertebral disc degeneration (IDD) is still unclear. It has been shown that the pathological process of IDD is most closely related to inflammation of nucleus pulposus cells (NPCs), in which inflammatory factors play an important role. Exosomes are the main paracrine mediators and are microvesicles with biological functions similar to those of the cells from which they are derived. Studies have shown that bone mesenchymal stem cells (BMSCs) can inhibit apoptosis of NPCs by sending exosomes as anti-inflammatory and antioxidant, which has been proved to be effective on IDD. However, the specific mechanism of inhibiting apoptosis of NPCs is still unclear. In our study, BMSC-derived exosomes (BMSC-Exo) were isolated from the BMSC culture medium, and their antiapoptotic effects were evaluated in cells and rat models to explore the possible mechanisms. We observed that BMSC-Exo promotes autophagy in NPCs and inhibits the release of inflammatory factors such as IL-1*β* and TNF-*α* in LPS-treated NPCs and inhibits apoptosis in NPCs. Further studies showed that BMSC-Exo inhibited the Akt-mTOR pathway. Intramuscular injection of BMSC-Exo alleviates disc degeneration in rat IDD models. In conclusion, our results suggest that BMSC-Exo can reduce NPC apoptosis and alleviate IDD by promoting autophagy by inhibiting the Akt-mTOR pathway. Our study confers a promising therapeutic strategy for IDD.

## 1. Introduction

There are many causes of low back pain, but intervertebral disc degeneration (IDD) is the main cause and has become one of the most common diseases that seriously affect human health [1]. Currently, the main treatments for IDD include conservative treatment and surgical treatments such as removal of nucleus pulposus, which are aimed at treating symptoms, but there is no effective radical cure for IDD [2], which is largely due to the fact that its current pathogenesis is still poorly defined and known effective therapeutic targets are fewer. Therefore, it is of great significance to further explore the regulatory mechanism of IDD and find effective interventions for its prevention. Autophagy, as a hot topic in the field of biology, is believed to be involved in the occurrence and development of various degenerative diseases. It is currently believed that autophagy plays an essential role in the repair, degeneration, and death of intervertebral disc cells [3–5]. Related studies have found that autophagy plays a role like a two-edged sword during degeneration. On the one hand, normal bodies have a basal level of autophagy, which is of great significance for the renewal and repair of cells themselves [6–8]. On the other hand, excessive autophagy may lead to cell death, and the control of excessive autophagy in nucleus pulposus cells (NPCs) can reduce cell apoptosis and thus delay the progression of IDD [9]. Therefore, it is extremely meaningful to determine how to regulate disc autophagy and exert its benefits to limit its disadvantages.

Regenerative medicine with stem cell transplantation as the main therapy has gradually emerged in recent decades. Bone mesenchymal stem cells (BMSCs) can make direct contact with the NPCs and promote their proliferation through paracrine [10]. It was found that BMSCs could inhibit NPC apoptosis and relieve IDD by delivering exosomes as an anti-inflammatory and antioxidant agent [11]. However, the specific mechanism of bone mesenchymal stem cell-derived exosomes (BMSC-Exo) in inhibiting NPC apoptosis is still unclear. It has been found that the knockdown of the nuclear factor kappa-B (NF-*κ*B) promotes cellular autophagy through the AKT/mTOR pathway and that the autophagy caused by NF-*κ*B inhibition plays a protective role in the NPCs degeneration induced by lipopolysaccharide (LPS) [12]. Our previous study also found that BMSC-Exo inhibited NPC apoptosis by inhibiting the NF-*κ*B signaling pathway [13]. We, therefore, hypothesized that BMSC-Exo is likely to promote autophagy of NPCs through the AKT/mTOR pathway to reduce cellular inflammation and subsequently inhibit NPCs' apoptosis. This study intends to explore the possibility of BMSC-Exo regulating NPC autophagy to inhibit apoptosis and its specific mechanism, so as to provide new ideas for the treatment of IDD.

## 2. Material and Methods

### 2.1. Chemicals and Reagents

The chemicals and reagents are as follows: IMDM cell medium, F12 cell medium (Gibco); osteogenic/adipogenic/chondrogenic induced differentiation medium (Suzhou Syagen); II-type collagenase, LPS, 3-MA, IGF-1Triton, and X-100 Tween (Sigma); Rabbit type II collagen primary antibody, rabbit Cleaved Caspase-3 primary antibody, rabbit Bax primary antibody, rabbit LC3 primary antibody, rabbit Beclin-1 primary antibody, rabbit *β*-actin primary antibody, rabbit p-Akt primary antibody, rabbit Akt primary antibody, rabbit p-mTOR primary antibody, rabbit mTOR primary antibody, HRP-labeled goat anti-rabbit secondary antibody, rabbit CD45 primary antibody, rabbit CD34 primary antibody, rabbit CD29 primary antibody, rabbit CD90 primary antibody, rabbit CD63 primary antibody, rabbit CD9 primary antibody, Rabbit GAPDH primary antibody, and rabbit TSG101 primary antibody (Abcam); TBS buffer, PBS buffer (Biosharp); Exosome Isolation Kit (Life technologies); primary antibody dilutions, secondary antibody dilutions, Trizol Lysate, ECL kit, PKH67 Staining Kit, BCA Protein Quantitative Kit, Annexin V-APC/PI Apoptosis Detection Kit, DAPI staining solution, RIPA Lysate (Medium), and PMSF (Beyotime, Jiangsu); 5 All-In-One RT MasterMix, SYBR Green Supermix (Abm), PAGE Gel Rapid Preparation Kit (12.5%) (Shanghai Epizyme); TNF-*α* ELISA Detection Kit, IL-1*β* ELISA Detection Kit, and Shanghai Enzyme Link.

### 2.2. Experimental Animals

Eight SD male SPF rats (aged 6-8 weeks) weighing 200 ± 20 g, were purchased from Zhaoyan (Suzhou) New Drug Research Center Co., Ltd., License No. SCXK (Suzhou) 2018-0016, Qualification Certificate No. 202008469. The protocol was approved by the animal committee of the First Affiliated Hospital of Soochow University (approval number 353/2020). The experimental rats were adaptively fed in a mode of light and day alternation (light time: 07:00-18:00), with a temperature of about 25°C, the humidity of about 50%, and good ventilation, as well as free diet and drinking water.

### 2.3. Cell Isolation and Culture

BMSCs were isolated from 6-8-week-old SD male rats and cultured as the following description. Briefly, the bone marrow was separated from the femurs and tibias of rats by flushing with a serum-free culture medium (IMDM; Gibco, USA). The cells were lysed in the red blood cell lysate and cultured in IMDM (containing 10% FBS) at 37°C, humidity 95%-100% with 5% CO_2_. First change of medium was performed after 24-48 h, at the frequency of once every 2-3 d later. The cells were passaged and divided when they grew to about 80%-90% filling in the incubator. BMSCs used in this study were P4 generation cells.

The nucleus pulposus tissue was fragmented and digested for 180 min in the type II collagenase; the tissue residue was filtered by 100 *μ*m filter and centrifuged at 4°C for 5 min, with a radius of 1000 r/min; and the supernatant was removed. F12 medium was blown and mixed uniformly, repeated 3 times, inoculated according to 1 × 10^6^ cells per incubator for static culture, and passaged at the right time. All the NPCs used in this experiment were P3-generation cells. The cells were identified by toluidine blue staining and type II collagen immunocytochemical staining.

### 2.4. Evaluation of the Mutipotency of BMSCs

P4-generation BMSCs were inoculated with 6-orifice plates at 2 × 10^4^/well density, which was divided into adipogenic induction, osteogenic induction, and chondrogenic induction, respectively. BMSCs were cultured in the following medium types: (1) adipogenic differentiation medium containing high-glucose DMEM, 10% FBS, 0.1 mmol/L 3-isobutyl-1-methylxanthine, 10 *μ*g/ml insulin, 10 nM dexamethasone, 50 *μ*g/ml indomethacin, 100 U/ml streptomycin, and 100 U/ml penicillin; (2) osteogenic differentiation medium containing high-glucose DMEM, 10% FBS, 50 *μ*g/ml ascorbic acid, 10 mM *β*-glycerophosphate, 10 nM dexamethasone, 100 U/ml streptomycin, and 100 U/ml penicillin; and (3) chondrogenic differentiation medium containing high-glucose DMEM, 50 *μ*g/ml ascorbic acid, 100 nM dexamethasone, 1 mM sodium pyruvate, 40 *μ*g/ml proline, 100 U/ml streptomycin, 100 U/ml penicillin, 10 ng/ml TGF*β*3, ITS+premix (final concentrations, 6.25 *μ*g/ml bovine insulin, 6.25 *μ*g/ml transferrin, 6.25 *μ*g/ml selenous acid, 5.33 *μ*g/ml linoleic acid, and 1.25 mg/ml bovine serum albumin). The induction medium was changed every 3 days. At day 14, cells were fixed and stained with Oil Red O for adipocytes, Alizarin Red for osteocytes, and Alcian Blue for chondrocytes.

### 2.5. Characterization of BMSCs by Flow Cytometry

Flow cytometry was utilized to determine the stemness features of BMSCs by analysis of specific cell surface markers. After being trypsinized, the cells were resuspended in 0.5 ml phosphate-buffered saline (PBS) and incubated for 30-40 min at 4°C with fluorescently labeled antibody (blank control tube without antibody) and resuspended in 0.3 ml PBS and then analyzed.

### 2.6. Extraction and Identification of BMSC-Exo

When P4-generation BMSCs were grown up to 80%, serum-free IMDM was cultured for 48 h, and after collecting the supernatant, the exocrine was extracted by ultra-high-speed centrifugation. Briefly, culture supernatant was collected and centrifuged at 300 × g for 10 min at 4°C and retain the supernatant, continue 2000 × g centrifugation at the same temperature for 20 min and retain the supernatant, continue 10000 × g centrifugation at the same temperature for 30 min and retain the supernatant, and continue 100000 × g centrifugation at the same temperature for 70 min; the precipitation obtained is exosomes, diluted with 1 × PBS, and stored in a refrigerator at -80°C. BMSC-Exo morphology was observed by a transmission electron microscope. A small amount of exosome samples were taken in three portions for dilution, and 1, 3, and 7 times volumes of 1 × PBS were added, respectively. Then, drop 20 *μ*l of each diluted sample onto the surface of copper mesh covered with carbon film, let it stand for 60-90 s, use filter paper to absorb the excess samples, and let it stand for drying. Then, 10 *μ*l of uranium acetate was added to each sample for staining under dark conditions. After 1 min, the residual dye was absorbed by filter paper and dried at room temperature. The staining was observed under TEM and photographed. The distribution of exosome particle diameter data was detected by a nanoparticle tracking analyzer. Exosomes obtained after centrifugation were diluted with 1 × PBS (1 : 500). The pipetting gun absorbed 200 *μ*l, the instrument was used for corresponding predetection, and the concentration was adjusted to the appropriate range. The exosomes diluted to an appropriate concentration were detected by the computer, and the data distribution diagram of exosome particle diameter was obtained. BMSCs and exosome surface marker proteins were detected and identified by Western blot.

### 2.7. Exosome Labeling and Cellular Uptake

After taking the mother solution of the PKH67 staining kit at room temperature, the exosomes containing 100 *μ*g protein were added and incubated for 15 min at room temperature. 10 ml 1 × PBS was added before adding half of the reagent from the exosome extraction kit, mixing and resting it at 4°C for 1 h, then centrifuged at 10,000 × g for 30 min, and the precipitation was resuspended with 200 *μ*l 1 × PBS; then, PKH67-labeled exosomes were obtained. They were incubated with adherent NPCs for 48 h, fixed in paraformaldehyde (4%), and washed three times with PBST (1 × PBS + 0.1% Triton X-100) for 10 min each. 3 ml of 1% BSA was added to incubate for 30 min and washed with PBST (1 × PBS + 0.1% Tween) 3 times, 10 min each time. 3 mL DAPI staining solution at 0.5 *μ*g/ml was added and incubated for 10 min, washed 3 more times with PBST (1 × PBS + 0.1% Tween) or 10 min each, then photographed and observed under a fluorescence inverted microscope.

### 2.8. Detection of NPC Viability by CCK-8

The digested NPCs were inoculated in 96-orifice plate 2 × 10^4^ cells/orifice. 24 hours later, the cells were cultured in the medium containing different concentrations of LPS (0, 100, 200, 500, and 1000 *μ*g/ml) for 24 hours, with six replicates for each concentration. Each orifice was incubated with 10 *μ*l CCK-8 reagent for 60 min. The *A* value of each orifice was measured by a microplate reader at a wavelength of 450 nm, showing the vitality of NPCs. The cell survival rate is calculated as follows: cell viability = [(As − Ab)/(Ac − Ab)] × 100%, where As is a value of experimental orifices (culture medium containing nucleus pulposus cells, CCK-8, and LPS); Ac is LPS concentration = a value of cell orificeat 0 *μ*g/ml; Ab is a value of blank orifice (medium without nucleus pulposus cells, LPS, and CCK-8); andAb = 0.002in this experiment. The appropriate concentration of LPS was selected according to the results to induce apoptosis of NPCs.

### 2.9. Effect of Exosomes on Apoptosis and Autophagy in NPCs

#### 2.9.1. Establishment of NPC Inflammation Model

The specific groups are as follows: control group: normal cultured NPCs; model group: normally cultured NPCs were incubated with 2 ml of F12 complete medium containing 500 *μ*g/ml LPS for 48 h to establish the inflammatory model of NPCs; exosome treatment group: normally cultured NPCs were incubated with 2 ml of F12 complete medium containing LPS of 500 *μ*g/ml as well as BMSC-Exo 20 *μ*g for 48 h; autophagy inhibitory group: normally cultured NPCs were incubated with 2 ml of F12 complete medium containing LPS of 500 *μ*g/ml and 10 mM of autophagy inhibitor 3-methyladenine (3-MA) as well as BMSC-Exo 20 *μ*g for 48 h.

#### 2.9.2. Reverse Transcription-PCR

The cells were collected, and RNA was extracted to detect the concentration of extracted RNA. RNA was reverse transcribed to generate cDNA and amplified using this as a template. Set the extended parameter to 95°C, 40s, 95°C, 10s, 60°C, and 35 s, for a total of 30 cycles. The result data were analyzed using 7300 System SDS Software and the relative expression levels of type II collagen (COL2A1), Beclin-1, and IL-1*β* in nucleus pulposus cells for each group using reference GAPDH as a control, i.e., 2 ^-△△Ct^ value. 2^−△△Ct^ = (CT_Target genes of experimental group_ − CT_GAPDH of experimental group_) − (CT_target genes of Control group_ − CT_GAPDH of Control group_). The expression levels of type II collagen, Beclin-1, and IL-1*β* were detected with GAPDH as the internal reference. The primer sequence is shown in Table 1.

#### 2.9.3. Western Blot Analysis

The protein of nucleus pulposus cells in each group was extracted and quantified by the BCA method. Following the electrophoresis separation with 30 *μ*g protein samples, membrane transfer, membrane cutting, and sealing, as well as incubation with primary antibody and secondary antibody, were carried out and then exposed and photographed. Using actin as the internal reference, the gray value of each band was calculated to obtain the relative expression of each histone.

#### 2.9.4. Enzyme-Linked Immunosorbent Assay (ELISA)

The supernatants of each group were collected; centrifuged at room temperature, 500 × g, 5 min; and gently absorbed with a pipette gun, and the contents of IL-1*β* and TNF-*α* were measured according to the instructions of IL-1*β* and TNF-*α* ELISA kits.

### 2.10. BMSC-Exo Promotes Autophagy by Inhibiting the Akt-mTOR Pathway

NPCs were divided into the following groups: degeneration group: normally cultured NPCs were incubated with 2 ml of F12 complete medium containing 500 *μ*g/ml LPS for 48 h; exosome group: normally cultured NPCs were incubated with 2 ml of F12 complete medium containing LPS of 500 *μ*g/ml as well as BMSC-Exo 20 *μ*g for 48 h; pathway activation group: normally cultured NPCs were incubated with F12 complete medium at 20 ng/ml containing LPS of 500 *μ* g/ml and Akt-mTOR pathway insulin-like growth factor-1 (IGF-1) as well as BMSC-Exo 20 *μ*g for 48 h. Western blot detects Akt-mTOR pathway protein expression in NPCs.

### 2.11. Establishment of Animal Model

#### 2.11.1. Animals Were Divided into Three Groups

*Control group*: the animal model of IDD in rats was established by needle puncture of the annulus fibrosus. The specific method was as follows: healthy SPF male SD rats were anesthetized by intraperitoneal injection of 1% pentobarbital sodium solution at 0.4 mL/kg; they were placed in a prone position to disinfect the skeletons and tails. The No. 21 injection needle was used to puncture the intervertebral space vertically through the dorsal center for about 5 mm; the needle was rotated 360 degrees and stayed for 30 s. After disinfection, the rats were free to move and then, the animal model of intervertebral disc degeneration was established. A little saline was then injected into the site of the disc space.

*Exosome treatment group (exosome)*: exosomes were injected for 30 *μ*g into the exposed disc space in the rat IDD model.

*Pathway activation group (IGF-1)*: IGF-1 60 ng as well as 30 *μ*g of exosomes was injected into the site of the exposed disc space in the rat IDD model.

#### 2.11.2. Histological Examination

Rats were sacrificed after 8 weeks; Co9/10 disc tissue was taken and fixed with 10% neutral formaldehyde, paraffin-embedded after decalcification with 10% EDTA for 4 weeks, then sectioned, followed by HE staining and immunohistochemical staining for type II collagen; and then, nucleus pulposus tissue damage was observed under a microscope. The acquired images were collected on an image analysis system and analyzed with Image-Pro Plus 6.0 software.

#### 2.11.3. Inspection

An MRI examination was performed 4 weeks and 8 weeks after injection to assess the degree of disc degeneration. 1.5 T MRI (GE HDx, Milwaukee, WI, USA) was used to obtain sagittal T2-weighted images, on which the three groups of model animals were classified from grades I to V according to the modified Thompson score (I means normal; II means slightly decreased signal, but the high signal area decreased significantly; III means moderately decreased signal; and IV means significantly decreased signal intensity).

### 2.12. Statistical Analysis

The measurement data were expressed by mean ± standard deviation ($\overset{¯}{x}\pm S$), the comparison among multiple groups was analyzed by ANOVA analysis, a *t*-test was used for the comparison between two groups, and SPSS 20.0 software was used for statistical analysis of experimental data, and *P* < 0.05 was considered statistically significant.

## 3. Result

### 3.1. The Identification of Rat BMSCs, NPCs, and BMSC-Exo

**Figure 1:**
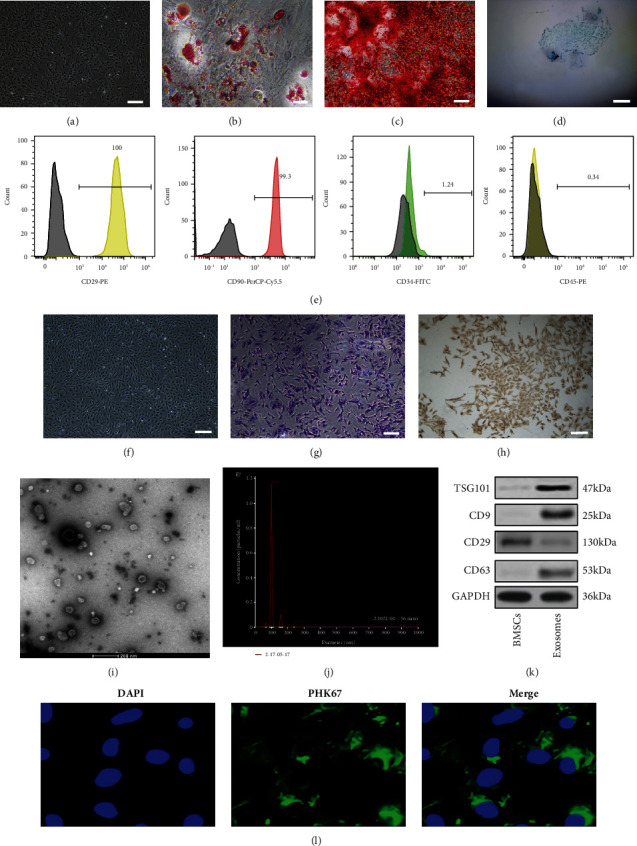
Morphology and characterization of BMSCs, NPCs, and BMSC-Exos: (a) P0-generation BMSC morphology (scale bar: 200 *μ*m); (b) Oil Red O staining (scale bar: 100 *μ*m); (c) Alizarin Red staining (scale bar: 100 *μ*m); (d) Alcian Blue staining (scale bar: 50 *μ*m), *n* = 3; (e) flow cytometric analysis of BMSC surface markers (CD29, CD90, CD34, and CD45); (f) NPC morphology (scale bar: 200 *μ*m); (g) toluidine blue staining of NPCs (scale bar: 100 *μ*m); (h) type II collagen immunochemical staining of NPCs (scale bar: 100 *μ*m); (i) transmission electron microscopy (TEM) image of BMSC-Exo (scale bar: 200 nm); (j) NanoSight tracking analysis (NTA) for the diameters of BMSC-Exo; (k) Western blot analysis of CD9, CD63, and TSG101, *n* = 3; (l) analysis of cellular exosome uptake by NPCs. *n* = 3. Scale bar: 25 *μ*m.

The BMSCs grew evenly in a long spindle-like morphology (Figure 1(a)) under light microscopy (×100). Oil Red O staining (Figure 1(b)), Alizarin Red staining (Figure 1(c)), and Alcian Blue staining (Figure 1(d)) showed that the BMSCs could differentiate into adipocytes, osteoblasts, or chondrocytes. Flow cytometry was used to detect the specific expression of CD90 and CD29 on the membrane surface of BMSCs, while the expression of CD34 and CD45 was not detected (Figure 1(e)). Primary NPCs were cultured for 14 d and the morphology changed to a long spindle containing secretory granules (Figure 1(f)). Toluidine blue-stained nuclei were blue and showed a slightly lighter extracellular matrix, indicating that cells can secrete glycosaminoglycans (Figure 1(g)). Type II collagen immunocytochemical staining showed that the cytoplasm was stained in brown-yellow, indicating type II collagen expression within the cytoplasm (Figure 1(h)). The BMSC-Exo was characterized via TEM, NTA, and Western blot. TEM data showed that exosomes had a cup-shaped structure with a diameter of about 100 nm (×50,000) (Figure 1(i)). NTA demonstrated that the diameter of exosomes was mostly 100 nm (Figure 1(j)). Western blot results showed that the maker proteins of the exosome membrane, CD9, CD63, and TSG101, were expressed in exosomes and CD29 in BMSCs (Figure 1(k)). The exosomes stained by PKH67 staining showed green fluorescence, and the nucleus in NPCs stained by DAPI staining showed blue fluorescence, through being codisplayed; the exosomes in NPCs showed green fluorescence in the nucleus pulposus cytoplasm, suggesting that the exosomes have the ability of membrane fusion and can be ingested by NPCs (Figure 1(l)).

### 3.2. BMSC-Exo Inhibits NPC Apoptosis by Promoting Autophagy

**Figure 2:**
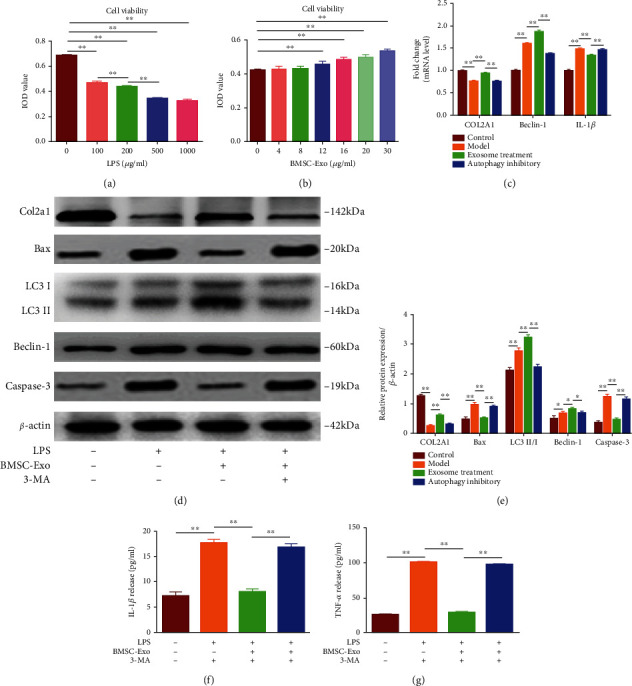
BMSC-Exo inhibits NPC apoptosis by promoting autophagy: (a) the integrated optical density (IOD) of NPCs treated with LPS gradient concentration; (b) IOD of NPCs treated with BMSC-Exo gradient concentration; (c) the mRNA expression of NPCs after incubation with LPS, BMSC-Exo, or 3-MA; (d–g) protein levels of autophagy and apoptosis-related protein (Col2a1, Baxter, LC3, Beclin-1, and Cleaved Caspase-3) in NPCs after being treated with LPS, BMSC-Exo, or 3-MA. *n* = 6. Data are presented as mean ± SD. Statistical analysis was performed with Student's *t*-test and one-way ANOVA followed by Tukey's multiple comparisons test. ^∗^*P* < 0.05; ^∗∗^*P* < 0.01.

To induce the apoptosis of NPCs, we cultured the NPCs with LPS gradient concentration (0, 100, 200, 500, and 1000 *μ*g/ml). The CCK-8 results showed that after being treated with 500 *μ*g/ml LPS, the integrated optical density (IOD) values decreased significantly (*P* < 0.01) (Figure 2(a)); we therefore established the apoptosis model by treating NPCs with 500 *μ*g/ml LPS. After being treated with BMSC-Exo gradient concentration (12, 16, 20, and 30 *μ*g/ml) for 48 h, the viability of NPCs increased gradually (Figure 2(b)). Compared with the control group, the mRNA expression of COL2A1 significantly decreased, while the expression of Beclin-1 and IL-1*β* significantly increased in the model group (treated with 500 *μ*g/ml LPS) (*P* < 0.01); compared with the model group, the mRNA expression of COL2A1 and Beclin-1 increased significantly, while the expression of IL-1*β* decreased in the exosome treatment group (treated with 500 *μ*g/ml LPS and 20 *μ*g/ml BMSC-Exo) (*P* < 0.01); compared with the exosome treatment group, the mRNA expression of COL2A1 and Beclin-1 decreased significantly, while the expression of IL-1*β* increased significantly in the autophagy inhibition group (treated with 500 *μ*g/ml LPS, 20 *μ*g/ml BMSC-Exo, and 10 mM 3-MA) (*P* < 0.01) (Figure 2(c)). Furthermore, we detected the autophagy and apoptosis-related protein (Col2a1, Baxter, LC3, Beclin-1, and Cleaved Caspase-3) expression with Western blot analysis and the release of IL-1*β* and TNF-*α* with ELISA, which was consistent with the mRNA expression (Figures 2(d)–2(g)). In summary, these results demonstrated that BMSC-Exo inhibits NPC apoptosis by promoting autophagy.

### 3.3. BMSC-Exo Promotes Autophagy by Inhibiting the Akt-mTOR Pathway

**Figure 3:**
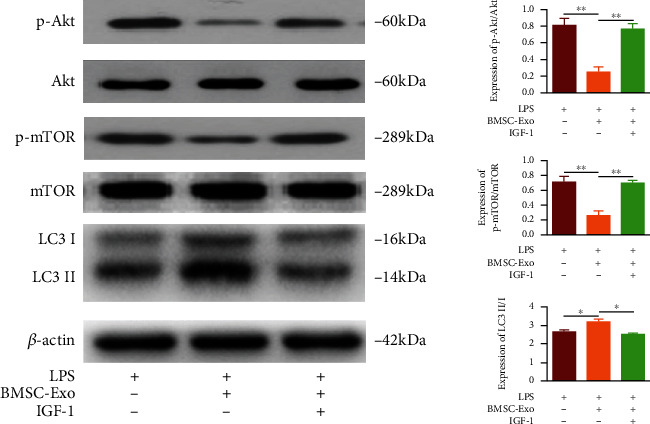
Western blot detects the relative expression changes of p-Akt, Akt, p-mTOR, mTOR, and LC3 in each group. *n* = 6. ^∗^*P* < 0.05; ^∗∗^*P* < 0.01.

To elucidate the mechanism by which exosomes promote autophagy, NPCs were divided into three groups: degeneration group, exosome group, and pathway activation group. The relative expressions of autophagy pathway-related proteins like p-Akt, p-mTOR, and LC3 II/I in NPCs were detected by Western-blot. The results showed that compared with the degeneration group, p-Akt/Akt and p-mTOR/mTOR were significantly reduced, and LC3 II/I was significantly increased in the exosome group. Compared with the exosome group, p-Akt/Akt and p-mTOR/mTOR were significantly increased, while LC3 II/I was significantly decreased in the pathway activation group (Figure 3(a)). Thus, exosomes could significantly reduce the relative expression of p-Akt and p-mTOR; however, when the Akt-mTOR pathway was activated, the levels of p-mTOR and p-Akt were recovered and autophagy levels were reduced, suggesting that exosomes facilitate autophagy by inhibiting the Akt-mTOR pathway in LPS-induced degeneration of NPCs.

### 3.4. BMSC-Exo Promotes Autophagy to Alleviate Disc Degeneration through Inhibition of the Akt-mTOR Pathway in the Rat IDD Model

**Figure 4:**
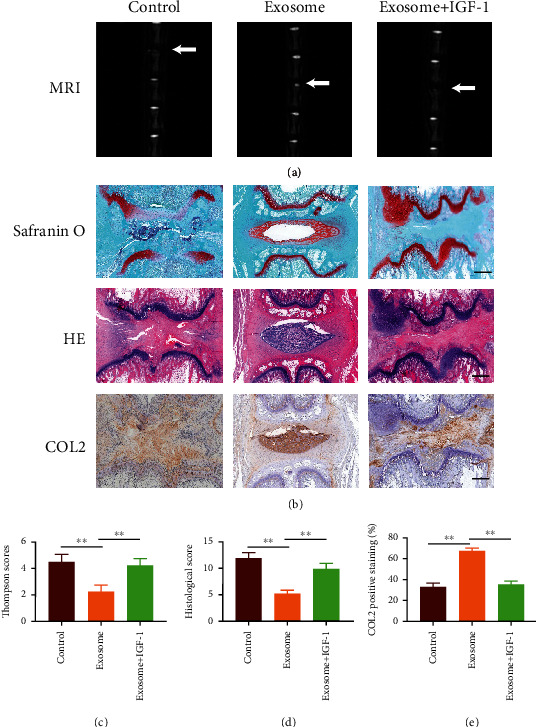
Results of the experiments in live animals. (a) MRI findings after injection of exosomes and IGF-1 into the caudal disc of a rat. (b) Histological analysis of HE and S/O staining discs. Immunohistochemical staining of type II collagen showed the presence of type II collagen in the exosome group, but little in the control and exosome+IGF-1 groups. Scale bar: 100 *μ*m. (c) Thompson scores of three groups. The score of the exosome group is lower than that of the other groups. (d) Histological score showed significant imaging improvement at week 8 of the therapy with exosome, but higher in the control and exosome+IGF-1 groups; (e) Immunohistochemical staining of type II collagen area (%). ^∗∗^*P* < 0.01.

In further studies, we established a rat model of intervertebral disc degeneration to observe the therapeutic effect of BMSC-Exo on the degeneration. MRI scans were performed on all of the rats. The results showed a significant decrease in the exosome group in terms of the T2-weighted phase of Thompson scores at the 8th week of treatment (*P* < 0.05) compared with the other two groups (Figures 4(a) and 4(c)). Staining in the control and pathway activation groups showed a reduction in the disc nucleus and the number of nucleus cell number and also a damage in disc annulus fibrosus. However, more nucleus pulposus cells and annulus fibrosus were intact in the exosome treatment group (Figures 4(b) and 4(d)). The function of the disc was determined by collagen II. Administration of exosomes resulted in increased expression of type II collagen compared to the control and pathway activation groups (Figure 4(e)). This suggests that exosomes can contribute to the recovery of the structure and function of disc degeneration and effectively promote the synthesis and secretion of the major matrix components in disc degeneration, which is achieved through the activation of the Akt-mTOR pathway.

## 4. Discussion

The pathogenesis of IDD is unknown, and evidence has been established that the pathological process of disc degeneration is strongly associated with increased oxidative stress, inflammatory factors, apoptosis, and increased matrix metalloproteinases [12]. The inflammation is most closely related to the NPCs, and the inflammatory factors play an essential role [14, 15]. Proinflammatory factors not only inhibit the synthesis of the extracellular matrix but also induce apoptosis, leading to a decrease in the number of the NPCs, which further aggravates IDD [12]. It has been shown that MSC-derived exosomes can inhibit the inflammatory response in the disc and thus reduce the apoptotic response of NPCs, but the mechanism is not clear. Therefore, it is necessary to investigate the mechanism of MSC-derived exosomes in the disc inflammatory response and apoptosis.

Exosomes are microvesicles measuring around 40–100 nm in diameter and contain a variety of biologically active substances [16]. Studies have shown that exosomes play an important role in cellular communication and metabolic regulation [17]. BMSC-Exo inhibits the activation of inflammatory mediators and reduces nucleus pulposus cell apoptosis [11]. Exosome separation and extraction methods predominantly include ultracentrifugation, polymer precipitation, ultrafiltration, immunoaffinity, and microfluidic separation technology. Ultracentrifugation is the “gold standard” and the most widely used [18]. The method was used to extract exosomes in this study. The International Society for Extracellular Vesicles stipulates that the identification of exosomes must include particle size, morphology, and protein molecular markers [19]. The extracted exosomes were identified by observing the particle size with a nanoparticle tracking analyzer and detecting surface markers with immunoblotting assays, and using immunofluorescence assay, it was confirmed that they could be ingested by NPCs. We found that the apoptosis in the exosome group was blindingly evident by flow cytometry, suggesting that BMSC-Exo can be ingested by NPCs and reduce their apoptosis.

LPS is an important proinflammatory factor in the human body that can induce strong inflammation in NPCs leading to apoptosis, reducing extracellular matrix synthesis, and plays an important role in IDD [12]. LPS can activate the inflammatory response to promote apoptosis [12]. Therefore, LPS was selected as the induction medium in our study; it was found in the CCK-8 experiment that the *A* value gradually decreased with the increase of LPS concentration, it was positively correlated with cell viability, and there was no significant difference in this value between the groups tested with LPS 500 *μ*g/ml and 1000 *μ*g/ml. Therefore, we used LPS 500 *μ*g/ml concentration to establish the cell apoptosis model and detect the apoptosis rate by flow cytometry; it was observed that LPS effectively induced the apoptosis of NPCs.

It was shown that disc degeneration could produce a large number of inflammatory factors, such as TNF-*α* and IL-1*β* [20]. As an initiation factor of inflammation, TNF-*α* can stimulate the expression of other inflammatory factors, and there is also a synergy between TNF-*α* and the stimulated inflammatory factors, such as IL-1*β*, showing a “positive feedback” effect [21]. It was found that the extracellular matrix degradation was increased after stimulation of NPCs by IL-1*β*, advancing the development of IDD [22]. Therefore, the inflammatory state of the cells can be understood by the detection of TNF-*α* and IL-1*β*. In this experiment, we examined the expression of IL-1*β* mRNA by RT-qPCR and found that the expression of IL-1*β* mRNA was significantly increased in NPCs after LPS-induced degeneration, and that of IL-1*β* mRNA was significantly decreased after the treatment with exosome. The detection of the concentrations of TNF-*α* and IL-1*β* concentrations in the supernatant for each group by ELISA showed that the concentrations in the model group increased apparently compared with the those in the control group, and those in the exosome group were significantly lower compared with those the model group. All of the results suggested that LPS promoted the inflammatory response of NPCs but exosomes inhibited it.

Autophagy, which is a key adaptive response, is a cellular self-protective process that can degrade and recycle intracellular damaged organelles and proteins [23]. It plays a key role in the pathogenesis of degenerative diseases (such as osteoarthritis and Alzheimer disease) [24]. Xu et al. found that autophagy attenuated the catabolic effect of NPCs in rats [25]. Therefore, we hypothesized that the inhibitory and antiapoptotic effects of the MSC-derived exosome may also be linked to autophagy.

LC3 protein is known to be important throughout autophagy. In the process of autophagy, the newly synthesized carbon terminal of LC3 is hydrolyzed by cysteine protease to generate LC3I. With the progression of the autophagy process, LC3I covalently binds phosphatidylethanolamine to form LC3II catalyzed by a ubiquitin-like reaction enzyme [23]. The ratio of LC3II/LC3I, as the end product of autophagy, is positively associated with the level of autophagy. Beclin-1 was the first identified mammalian autophagy-related gene, and it is considered a positive regulatory molecule of autophagy [24]. Therefore, the occurrence of autophagy can be reflected by detecting the content of LC3II/LC3I and Beclin-1. By measuring the Beclin-1 mRNA expression using RT-qPCR, it was found that the expression in the model group was significantly higher than that of the control group, and the Beclin-1 mRNA expression was further increased in the exosome group compared with the model group. Similar results were found in the detection of protein expression of LC3II/LC3I and Beclin-1 in each group by Western blot. The protein expression of LC3II/LC3I and Beclin-1 in the model group was increased significantly compared with that in the control group, whereas the expression was further increased in the exosome group compared to the model group. All of the results indicated that exosomes could promote autophagy in nucleus pulposus cells.

To investigate the relationship between the antiapoptotic effect of BMSC-Exo on nucleus pulposus cells and autophagy, we employed the autophagy pathway inhibitor 3-MA which can prevent the formation of autophagosome by inhibiting phosphoinositide 3-kinase (PI3K) and thus fundamentally inhibit autophagy from the source. We examined the expression of IL-1*β* mRNA by RT-qPCR after inhibiting autophagy using 3-MA and found that the expression of IL-1*β* mRNA increased again. However, the TNF-*α* and IL-1*β* content of supernatants in each group also found that the TNF-*α* and IL-1*β* contents were significantly increased in the autophagy inhibition group compared with the exosome group. All the results suggest that the inhibitory effect of exosomes is reversed after autophagy is inhibited, suggesting that exosomes may produce the effect of inhibiting inflammation by promoting autophagy. The flow cytometry showed that exosomes could inhibit apoptosis of nucleus pulposus cells due to LPS, while the rate of apoptosis increased again after inhibition of autophagy using 3-MA. Additionally, we examined the nucleus myelocytes Bax, Cleaved Caspase-3, and type II collagen by Western blot. Bax is an important factor in the regulation of apoptosis and mainly resides in the cytoplasm, which mediates the release of downstream apoptotic molecules and triggers apoptosis [26, 27]. The apoptosis may occur when Bax expression is upregulated [28, 29]. Caspase-3 is a key molecule for performing apoptosis, and as an activated form of Caspase-3, the formation of Cleaved Caspase-3 marks irreversible apoptosis [30]. Type II collagen is an important component of the disc, and it is found that this collagen in the disc is significantly decreased in IDD patients [31]. Therefore, the expression of type II collagen in nucleus pulposus cells is positively correlated with the degree of degeneration. Results are shown: the expression of Cleaved Caspase-3, Bax, and type II collagen was decreased significantly in the model group compared with the control group. In comparison with the model group, the expression of type II collagen was increased and that of Cleaved Caspase-3 and Bax decreased significantly in the exosome group. Compared with the exosome group, Cleaved Caspase-3 and Bax showed an evident increase, and that of type II collagen was significantly decreased in the autophagy inhibition group. In this experiment, we also investigated the expression of type II collagen by RT-qPCR experiments for each group, and the results showed that the expression of mRNA in the model group was significantly reduced compared with that in the control group, whereas that of the mRNA in the exosome group of collagen mRNA expression was significantly increased compared with that in the model group, and that of the collagen was decreased in the autophagy inhibition group as compared to the exosome group. All the results indicate that exosomes can inhibit nucleus pulposus cell apoptosis, but its antiapoptotic effect is basically reversed after autophagy is inhibited, suggesting that BMSC-Exo may exert an antiapoptotic effect by promoting autophagy.

Numerous studies have shown that the Akt-mTOR signaling pathway is an important pathway to regulate the biological functions of various cells [32–34], including the regulation of the biological function of NPCs [35–37]. The Akt-mTOR signaling pathway is also involved in the progression of various human degenerative diseases, including the degeneration of nucleus pulposus cells [38]. As a typical autophagy signaling pathway, the Akt-mTOR pathway shows a negative role in the regulation of autophagy. We therefore hypothesized that BMSC-Exo would probably produce anti-inflammatory and antiapoptotic effects by inhibiting Akt-mTOR signaling to promote the autophagy of nucleus pulposus cells.

To prove this hypothesis, we applied the pathway activator IGF-1 on degenerative nucleus pulposus cells after exosome treatment. We examined the activation of the Akt-mTOR pathway by Western blot to test the phosphorylation-activated mTOR (p-mTOR) and phosphorylation-activated Akt (p-Akt) and investigated the changes in LC3/LC3I expression as well as the activation of autophagy. It was found that the autophagy protein LC3II/LC3I increased markedly and the pathway proteins p-mTOR and p-Akt showed a decline in the exosome group, while the expression of autophagy-related protein LC3II/LC3I was significantly decreased in the pathway activation group, and the expressions of p-Akt and p-mTOR protein were significantly higher than the exosome group (*P* < 0.05), suggesting that the Akt-mTOR signaling pathway was involved in the exosome-regulated autophagy process in nucleus pulposus cells.

**Figure 5:**
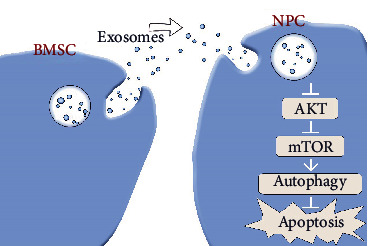
MSC-derived exosomes mitigate apoptosis against LPS-induced apoptosis by promoting nucleus pulposus cell autophagy, which is achieved through the Akt-mTOR signaling pathway.

Taken together, MSC-derived exosomes mitigate apoptosis against LPS-induced apoptosis by promoting nucleus pulposus cell autophagy, which is achieved through the Akt-mTOR signaling pathway (Figure 5). This study reveals the likely mechanism of exosomes alleviating LPS-induced inflammatory damage and apoptosis, which will provide a new direction for the treatment of IDD. However, it should be noted that all the current studies on exosomes are still in the basic research stage, and there are still many problems to be solved. The first thing is that there are certain differences between people and animals. Secondly, exosomes cannot be mass-produced, which also limits their clinical application [39]. Additionally, exosomes isolated from biofluid are impure and highly variable. Moreover, subsets of exosomes and the specific mechanisms underlying their effects on receptor cells remain poorly defined. Therefore, despite some achievements made in the treatment of IDD with exosomes, there is still a long way to go before it can be put into clinical application. It is believed that with the progress of the research, the treatment with exosomes will eventually become a new regimen for the clinical treatment of IDD.

## Figures and Tables

**Table 1:** Oligonucleotide primers used for RT-qPCR.

Genes	Forward primer	Reverse primer	Annealing (°C)
GAPDH	CTTCTCTTGTGACAAAGTGGA	TTAGCGGGATCTCGCTC	60
COL2A1	CCTGAAACTCTGCCACCCAG	GTTCTTCCGAGGCACAGTCG	60
Beclin-1	GTTCTCTCAGTTGCCTTTC	AGCTGTAACCTGTCGCCGAGTCCC	60
IL-1*β*	CCGGAGTCTGACTGGAAAGCC	GCATACAGGAAGTCGGCCTCC	60

## Data Availability

The data used to support the findings of this study are available from the corresponding author upon request.
